# Characterization and Outcome of Two Pediatric Intensive Care Units with Different Resources

**DOI:** 10.1155/2020/5171790

**Published:** 2020-03-17

**Authors:** Rania G. Abdelatif, Montaser M. Mohammed, Ramadan A. Mahmoud, Mohamed A. M. Bakheet, Masafumi Gima, Satoshi Nakagawa

**Affiliations:** ^1^Department of Pediatrics, Faculty of Medicine, Sohag University, Sohag 82524, Egypt; ^2^Division of Critical Care Medicine, National Center for Child Health and Development, Tokyo 157-8535, Japan

## Abstract

**Background:**

The pediatric intensive care units (PICUs) in developing countries have a higher mortality outcome due to a wide variety of causes. Identifying differences in the structure, patient characteristics, and outcome between PICUs with different resources may add evidence to the need for incorporating more PICUs with limited resources in the contemporary critical care research to improve the care provided for severely ill children.

**Methods:**

A retrospective study was conducted at Egyptian and Japanese PICUs as examples of resource-limited and resource-rich units, respectively. We collected and compared data of nonsurgical patients admitted between March 2018 and February 2019, including the patients' demographics, diagnosis, PICU length of stay, outcome, predicted risk of mortality using pediatric index of mortality-2 (PIM-2), and functional neurological status using the Pediatric Cerebral Performance Category (PCPC) scale.

**Results:**

The Egyptian unit had a lower number of beds with a higher number of annual admission/bed than the Japanese unit. There was a shortage in the number of the skilled staff at the Egyptian unit. Nurse : patient ratios in both units were only similar at the nighttime (1 : 2). Most of the basic equipment and supplies were available at the Egyptian unit. Both actual and PIM-2 predicted mortalities were markedly higher for patients admitted to the Egyptian unit, and the mortality was significantly associated with age, severe sepsis, and PIM-2. The length of stay was shorter at the Egyptian unit.

**Conclusion:**

The inadequate structure and the burden of more severely ill children at the Egyptian unit appear to be the most important causes behind the higher mortality at this unit. Increasing the number of qualified staff and providing cost-effective equipment may help in improving the mortality outcome and the quality of care.

## 1. Introduction

The pediatric intensive care unit (PICU) is a specialized unit designed primarily to provide qualified care for critically ill children that extends beyond its walls to include emergency department, wards, and prehospital settings [1, 2]. Since it was developed in the early 1960s, the PICU has a significant role in the reduction of childhood mortality in the developed world [3]. The last decade has also witnessed marked progress in advancing pediatric intensive care to the developing world [1]. However, many PICUs in low- and middle-income countries, where there is a higher percentage of pediatric population, still require a higher number of qualified health care staff as well as rapid access to necessary medication, supplies, and equipment to participate effectively in reducing childhood mortality [4].

Egypt is one of the lower-middle-income countries, according to the World Bank classification, where children represent the highest percentage of the population (33.5% were below 15 years old in 2016) [5, 6]. According to the reported estimates, the under-5 child mortality had been remarkably decreased from 86 to 21 per 1000 live births from 1990 to 2012 representing 75.4% drop [7]. However, this improvement should be maintained through continued organized policies that address and overcome the challenges in the context of limited resources. Hence, the information about the characteristics and outcome of patients admitted to the PICUs is valuable from health policy perspectives [8].

On the contrary, Japan, as an example of high-income countries, has a similar number of population as Egypt but with different age distribution (12.9% of the population were below 15 years old in 2016) [6, 9]. It had under-5 mortality of about 3 per 1000 live births in 2017 [6, 10]. It has a highly qualified health care system, especially for children [10].

Few international studies were conducted to globally evaluate pediatric critical services. Furthermore, researches from developing countries represent only small fraction of the whole critical care research. Incorporation of evidence about pediatric critical care services from different regions of the world is crucial to apply real beneficial care for children [8].

## 2. Main Study Objectives

The present study was conducted to investigate the extent of differences in the structure in terms of human and physical resources in two PICUs in Egypt and Japan, as well as, comparing their outcomes and factors associated with mortality. This provides useful information and suggests important interventions for improving the outcome not only at this particular Egyptian PICU but also at similar PICUs in Egypt and other developing countries.

## 3. Patients and Methods

### 3.1. Study Design and Setting

We conducted this retrospective study at two different PICUs between March 1, 2018, and February 28, 2019. The first one is the PICU located at Sohag University Hospital (SUH), Sohag, Egypt. SUH is a tertiary care university-affiliated hospital that serves Sohag Governorate with about 5 million population. The second one is the PICU located at the National Center for Child Health and Development (NCCHD), Tokyo, Japan. NCCHD is a tertiary children's hospital that serves for approximately 4 million of the population (a third of the total Tokyo's population).

The differences in human and physical resources were compared between both units in terms of number and availability. This included the beds, admissions, skilled staff, equipment/supplies, and drugs. The usage of the available therapeutic and invasive monitoring modalities was recorded for each patient.

### 3.2. Patients

We included patients 0–14 years old admitted at both units. Those who were admitted for postoperative care and those stayed less than 6 hours in the PICU were excluded.

## 4. Methods

We collected the following data from the patients' medical records: patients' demographics (age and gender), previous pathological conditions, presence or absence of cardiac arrest, main system involvement on admission, the clinical diagnosis on discharge, and the PICU length of stay in days (LOS). Risk of mortality was estimated using pediatric index of mortality-2 (PIM-2) and was calculated using the logistic regression equation [11]. The resident doctors at the emergency unit calculated this score during the first hour of admission followed by rapid transfer of the patients to the PICU. The functional neurological status was assessed by PICU physicians using the Pediatric Cerebral Performance Category (PCPC) scale as a baseline (on admission) and at the PICU discharge [12].

This study was approved by the Research Ethics Committee of the Faculty of Medicine, Sohag University and that of NCCHD.

### 4.1. Statistical Analysis

We analyzed the data using the Statistical Package for Social Science Software (SPSS) program version 16.0 IBM. Descriptive statistics were presented as frequencies and percentages for qualitative data. Pearson's chi-square test or Fisher's exact test were used to compare proportions. Quantitative data were reported in terms of median and interquartile range (IQR) due to their nonnormal distribution. Therefore, comparison between groups was made by the nonparametric MannWhitney *U* test. All tests were two-tailed, and *p*-value of less than 0.05 was considered statistically significant.

## 5. Results

### 5.1. The PICU Structure

The difference in the structure between the two units is highlighted in Table 1. The attending staff were more in terms of number and pediatric critical care qualification at the NCCHD PICU. Moreover, allied health care workers as pharmacists and physiotherapists were not available at the SUH PICU. Nurses were trained at both units to provide a valuable role in patient evaluation and monitoring, medication administration, and communication with other health care providers as well as patients' families. However, nurses at the Japanese unit were more trained in taking care of critically ill patients. The nurse : patient ratio was only similar at the nighttime 1 : 2. At the daytime, it was 1 : 1 at the NCCHD PICU compared to 1 : 2 at the SUH PICU. Also, the shifts were longer (12 hours) at the SUH PICU due to lower number of nurses. The availability of essential equipment, supplies, and drugs was comparable at both units. However, more technologically advanced equipment was present at the NCCHD PICU. The utility of some of these monitoring and life-supportive modalities is illustrated in Table 2. Additionally, electronic medical records of the patients were only available at the NCCHD PICU.

### 5.2. Patient Characteristics

**Figure 1:**
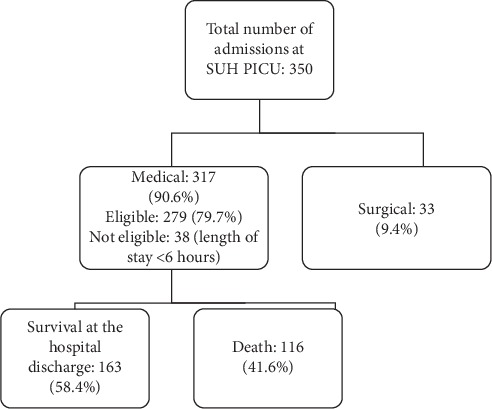
The flow chart of the study at the Sohag University Hospital Pediatric Intensive Care Unit (SUH PICU).

**Figure 2:**
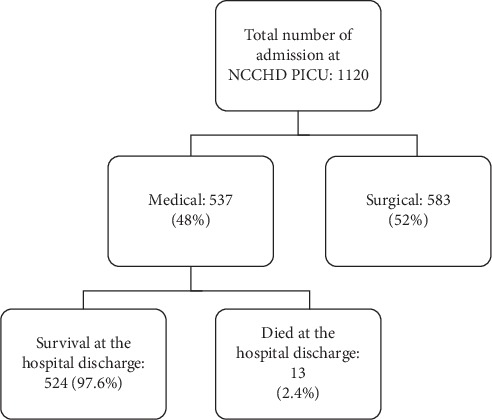
The flow chart of the study at the National Center for Child Health and Development Pediatric Intensive Care Unit (NCCHD PICU).

The patients' demographics of the two units were different, as the SUH PICU mainly had medical patients while NCCHD's patients were half postoperative and half medical. Postoperative patients from both units were excluded to focus on only the medical patients. The mortality was markedly higher at the SUH PICU than at the NCCHD PICU (Figures 1 and 2).

Table 3 summarizes patients' characteristics in SUH and NCCHD PICUs. In Table 4, the mortality outcome is compared between both units. Statistically significant differences were related to the age (*P* value: 0.024), PIM-2 (*P* value: <0.0001), and severe sepsis (*P* value: 0.014). Twenty patients (38% of cardiovascular deaths at the SUH PICU) had congenital heart diseases, whereas there were almost no deaths from congenital heart diseases at the NCCHD PICU.

## 6. Discussion

To the best of the authors' knowledge, this study is considered one of the few studies that highlighted the difference in the structure, patient characteristics, and mortality outcomes in two PICUs located at two regions of the world with different resources. This may add evidence to the need for incorporating more PICUs with limited resources in the contemporary critical care research to improve the care provided for severely ill children.

The structure of any PICU should have four basic components, according to Dr. Paul Farmer, which are (1) staff: properly trained health care professionals; (2) stuff: appropriate medical equipment; (3) space: a clean environment for patients; and (4) systems: the infrastructure and logistical organization to provide the services [4, 13, 14]. It is obviously understood that the mere presence of an intensive care unit does not guarantee a better outcome; the mortality rates can be as high as 50–58% in some PICUs in the developing world as most of the care is provided by personnel with poor pediatric critical care training [15, 16]. Therefore, the lack of adequate number and training of the staff members at the SUH PICU might contribute to the higher mortality. The nurse : patient ratios at both units follow the standard recommendation of being at least 1 : 2 [4]. However, the shifts were longer at the SUH PICU due to the shortage in the number of available nurses. In addition to staffing, adequate number of medical equipment is essential in pediatric critical care. According to a web-based survey conducted in 2014 on the resources of the pediatric critical care worldwide, the SUH PICU seems to have most of the basic equipment and drugs included in this survey [8]. Nevertheless, there is a lack of some important supplies and life-support equipment which are particularly needed at the SUH PICU as many patients were admitted with respiratory failure and shock in whom these modalities are essential in monitoring and life support. This survey also noticed a limited implementation of electronic health records in the PICUs of developing countries which was the condition in the SUH PICU [8].

The majority of patients at the SUH PICU were infants who also constituted the highest age-related mortality. This was also reported in different studies performed in many developing countries with higher population of children [17, 18]. This confirms the need to direct more resources to reduce the in-hospital mortality of this vulnerable age group.

In contrast to the NCCHD PICU, severe sepsis/septic shock was associated with a high percentage of admissions and reported fatality at the SUH PICU. The relatively higher percentage of sepsis and related mortality among the pediatric population is well documented in many developing countries where a combination of environmental and socioeconomic factors plays an important role in the spread of infections [19–21].

Additionally, patients with congenital heart diseases were associated with high mortality at the SUH PICU. This is because the majority of the admitted patients were infants in whom congenital heart diseases are common cause of mortality in Egypt; congenital anomalies were responsible for 21% of infant mortality in 2014 [7]. Although small or single cardiac defects can be corrected either surgically or via cardiac catheterization at SUH, these options are not possible for complex defects. This might partly contribute to the higher observed mortality from this disease. Conversely, surgical correction is usually performed for the majority of patients with congenital heart diseases in the early infancy at NCCHD. Another possible cause is the difference in the management attitude to the patients with hemodynamic instability. Invasive blood pressure monitoring is often carried out at the NCCHD PICU, but this is not done at the SUH PICU. This finding is supported by a study conducted in Thailand noticed that invasive blood pressure monitoring was nearly not performed for patients with shock, whereas this invasive modality was routinely performed in 99.9% of PICUs in the United States according to a national survey held in 2005 [22, 23].

According to the results of this study, the patients admitted at the SUH PICU had a higher PIM-2-based predicted mortality compared to those admitted at the NCCHD PICU. This is might be due to the variation in the access to the medical service. Despite the great national efforts to improve the health services all over Egypt, there are still areas in Upper Egypt with difficult access to the medical services [24]. Moreover, the prediction ability of PIM-2 differs markedly between the two units; it seems to underpredict mortality at the SUH PICU while it overestimates it at the NCCHD PICU. A similar situation is found in some studies conducted in various low- and high-income countries [19, 24, 25]. The limited human and physical resources in addition to the difference in the standards of care may contribute to the poor calibration of the score in the developing countries. [19, 24].

Despite the higher severity of illness at the SUH PICU, the median length of stay was nearly half than that at the NCCHD PICU. This is because many patients at the SUH PICU had to be early discharged to continue treatment at the intermediate care unit in order to receive more critical patients from the emergency room. This was supported by an Egyptian study which emphasized the importance of the existence of the intermediate care unit not only as a place for continuing care but also to provide more PICU beds for the other critically ill patients [17].

It was found that normal baseline cerebral function was present in more than half of patients admitted to both units. This was similar to a reference range of 54% to 84.3% [26, 27]. However, the percentage of survivors with normal PCPC was lower for those admitted to the SUH PICU. This may be explained by the higher severity of illness of the admitted patients. This finding is supported with a study performed by Volakli et al. who reported that the higher proportion of patients admitted with neurological emergencies and their higher severity of illness had resulted to lower proportions (21%) of patients discharged with normal cerebral function [28].

This study has some limitations which are important to mention: first, it compares a small with a large PICU in which there is a great discrepancy in the number of admissions and human and physical resources. Second, the Egyptian unit included in this study is a relatively small unit. Thus, the results cannot be generalized to all Egyptian PICUs. Third, there were different cohorts in the two units as the patients at the SUH PICU were mainly medical while nearly half of patients at the NCCHD PICU were surgical. Therefore, half of the patients at the NCCHD PICU had to be excluded to make the two groups comparable, which could affect the results. Finally, the study did not analyze in depth the difference in the treatment or management protocols at both units, which seems to be difficult to compare, but it is an extremely important determinant of the outcome.

## 7. Conclusion

In contrast to the NCCHD PICU, the SUH PICU had inadequate structure due to shortage in the number of qualified staff and advanced medical equipment. Both actual and PIM-predicted mortality was higher at the SUH PICU, particularly among infants with severe sepsis or congenital heart diseases. Increasing the number of qualified staff and providing cost-effective equipment may help in improving the mortality outcome and the quality of care.

## Figures and Tables

**Table 1:** The structure and human resources at SUH and NCCHD PICUs.

Variables	SUH PICU	NCCHD PICU
No. of beds	5	20
No. of annual admission	±320	±1100
No. of annual admission/bed	64	55
*Staff:*		
No. of attending staff	4 (pediatricians)	11 (pediatric intensivists)
No. of trainees	4	15
No. of nurses	23	70

NCCHD: National Center for Child Health and Development, Tokyo, Japan. SUH: Sohag University Hospital, Sohag, Egypt. PICUs: pediatric intensive care units.

**Table 2:** The utilization of different equipment and supplies at SUH and NCCHD PICUs.

Equipment/supplies	SUH (*n* = 279)	NCCHD (*n* = 537)
High-flow nasal cannula	0	177 (33%)
Noninvasive MV	0	25 (4.7%)
Invasive MV	80 (28.7%)	202 (37.6%)
High-frequency ventilation	0	1 (0.2%)
CRRT	0	26 (4.8%)
Arterial catheter	0	255 (47.5%)
Central venous catheter	54 (19.4%)	197 (36.7%)
Urinary catheter insertion	51 (18.3%)	223 (41.5%)
Inhaled nitric oxide	0	22 (4.1%)
Invasive ICP monitoring	0	5 (0.93%)
ECMO	0	8 (1.5%)

NCCHD: National Center for Child Health and Development, Tokyo, Japan. SUH: Sohag University Hospital, Sohag, Egypt. PICUs: pediatric intensive care units. CRRT: continuous renal replacement therapy. ECMO: extracorporeal membrane oxygenation. ICP: intracranial pressure. MV: mechanical ventilation.

**Table 3:** Patients' characteristics at SUH and NCCHD PICUs.

Patient characteristics'	SUH (*n* = 279)	NCCHD (*n* = 537)
Age in months^a^	7 (3–22)	24 (8–65)
Male/female^b^	153/126 (54.8/45.2)	307/230 (57.2/42.8)
PIM-2^a^	8.6 (1.6–58.8)	1.2 (0.9–4.8)
Length of stay in days^a^	3 (2–7)	6 (4–9)
*Main diagnostic categories* ^*b*^		
Cardiovascular disturbances	89 (31.9)	57 (10.6)
Respiratory distress/failure	50 (17.9)	208 (38.7)
Neurological disturbances	44 (15.8)	156 (29.1)
Postcardiac arrest	24 (8.6)	14 (2.6)
Others	72 (25.8)	102 (19)
Sepsis^b^	40 (14.3)	14 (2.6)
Comorbid conditions^b^	154 (55.2)	338 (62.9)
*Baseline PCPC* ^*b*^		
1 (normal)	158 (56.6)	368 (68.5)
2 (mild disability)	43 (15.4)	40 (7.4)
3 (moderate disability)	42 (15.1)	57 (10.6)
4 (severe disability)	35 (12.5)	72 (13.4)
5 (coma/vegetative state)	1 (0.4)	0
*Exit PCPC* ^*b*^		
1 (normal)	79 (28.3)	338 (62.9)
2 (mild disability)	34 (12.2)	48 (8.4)
3 (moderate disability)	30 (10.8)	56 (10.4)
4 (severe disability)	16 (5.7)	78 (14.5)
5 (coma/vegetative state)	4 (1.4)	4 (0.7)
6 (brain death/death)	116 (41.6)	13 (2.4)

(a) Median (interquartile range). (b) Number of patients (%). NCCHD: National Center for Child Health and Development, Tokyo, Japan. SUH: Sohag University Hospital, Sohag, Egypt. PICUs: pediatric intensive care units. PIM-2: pediatric index of mortality-2. PCPC: Pediatric Cerebral Performance Scale.

**Table 4:** Comparison of mortality between SUH and NCCHD PICUs.

Patient characteristics'	SUH (*n* = 116)	NCCHD (*n* = 13)	*P* value
Age in months^a^	2.3 (3–12)	23 (5–66)	0.024
Male/female^b^	62/54 (53.4/46.6)	5/8 (38.5/61.5)	0.305
PIM-2^a^	29.7 (9–94)	1 (0.7–4.8)	<0.0001
Length of stay in days^b^	3 (1–7)	4 (1.5–10)	0.191
*Main diagnostic categories* ^*b*^			
Cardiovascular disturbances	52 (44.8)	3 (23.1)	0.309
Respiratory distress/failure	10 (8.6)	1 (7.7)	
Neurological disturbances	22 (18.9)	2 (15.4)	
Postcardiac arrest	20 (17.2)	6 (46.2)	
Others	12 (10.3)	1 (7.7)	
Severe sepsis/septic shock^b^	33 (28.4)	0	0.014
Comorbid conditions^b^	69 (59.5)	9 (69.2)	0.460

(a) Median (interquartile range). (b) Number of patients (%). NCCHD: National Center for Child Health and Development, Tokyo, Japan. SUH: Sohag University Hospital, Sohag, Egypt. PICUs: pediatric intensive care units. PIM-2: pediatric index of mortality-2.

## Data Availability

The datasets generated and/or analyzed during the current study are not available publicly due to privacy and are available from the corresponding author upon reasonable request.
